# Prevalence and predictors of obstructive sleep apnea in patients with bipolar disorder: A systematic review and meta-analysis

**DOI:** 10.12669/pjms.41.1.10567

**Published:** 2025-01

**Authors:** Fangyan Zhu, Chenmin Cui, Kailong Gu

**Affiliations:** 1 Fangyan Zhu Department of Psychosomatic Disorders, Huzhou Third Municipal Hospital, The Affiliated Hospital of Huzhou University, Huzhou, Zhejiang Province 313000, China; 2 Chenmin Cui Department of Nephrology Huzhou Traditional Chinese Medicine Hospital Affiliated to Zhejiang Chinese Medical University, Huzhou, Zhejiang Province, 313000, China; 3 Kailong Gu Department of Geriatric Psychiatry, Huzhou Third Municipal Hospital, The Affiliated Hospital of Huzhou University, Huzhou, Zhejiang Province 313000, China

**Keywords:** Obstructive sleep apnea, Mental disorder, Major depressive disorder, Bipolar disorder, Comorbidity

## Abstract

**Background & Objective:**

Obstructive sleep apnea (OSA) has been increasingly recognized as a comorbidity in many psychiatric disorders, including bipolar disorder (BD). This study aimed to synthesize existing evidence to determine the frequency of OSA in patients diagnosed with BD and identify potential predictors of its occurrence.

**Methods:**

PubMed, Scopus, CENTRAL (Cochrane Central Register of Controlled Trials), and Google Scholar databases were searched for English-language papers published up from 1^st^ January 1960 to 31^st^ October 2023 that reported incidences of OSA in patients with BP and provided sufficient data for quantitative analysis. The Preferred Reporting Items for Systematic Reviews and Meta-Analyses (PRISMA) and Cochrane Handbook (version 5.1.0) guidelines were followed. Meta-regression analyses were done to identify predictors that correlate with the prevalence of obstructive sleep apnea.

**Results:**

The systematic review identified a total of 14 eligible studies. The pooled prevalence of OSA in individuals with BD was determined to be [24.55%] (95% confidence interval (CI): [17.25 to 32.63%, I^2^=99.57%]). Meta-regression analyses showed that male gender was associated with a higher prevalence of OSA, while body mass index (BMI) and mean age showed no significance in predicting OSA prevalence.

**Conclusion:**

Our results demonstrated higher incidence of OSA in patients with BD. Male BD patients had higher rate of OSA.

## INTRODUCTION

Bipolar disorder is a significant public health concern characterized by extreme mood swings, including depressive and manic episodes.^1^ Globally, the prevalence of bipolar disorder is estimated at around 1-2% in the general population, with some studies suggesting lifetime prevalence rates of 4-5%.^2^ Recent data indicate approximately 39.5 million cases worldwide as of 2019, reflecting a 59.3% increase since 1990. Annual incidence rates are approximately 3.4 million new cases, with higher rates reported in individuals aged 18-29 years. The prevalence is roughly equal between genders, though females may experience more depressive episodes, and males more manic episodes.^3^ The disorder causes profound impairment, with 82.9% of individuals experiencing severe functional limitations, making it one of the most disabling mental illnesses globally.^4^,^5^

The impact of BD extends beyond the symptoms and functional impairments linked to the disorder. Patients with BD are at an increased risk of various medical issues, and have 9-13 years shorter life span compared to the general population.^6^ The prevalence of obstructive sleep apnea (OSA) increases with age and obesity and is strongly linked to adverse health outcomes such as cardiovascular diseases, including stroke, coronary heart disease, and heart failure. Key risk factors include male gender, postmenopausal status, elevated BMI, and ages 40-70 years. Untreated OSA contributes to significant mortality and cardiovascular risks, yet it remains widely underdiagnosed, imposing substantial health and economic burdens globally.^7^,^8^

The impact of OSA on the quality of life may provide a partial explanation for the shortened life expectancy observed in patients with BD. Despite its obvious importance, the association of BD and OSA is still unclear. Six studies, incorporating clinical samples and population cohorts, reported varying prevalence rates of OSA in patients with BD (ranging from 2.9% to 69%), with discrepancies attributed to differences in sample definitions.^1^,^9^,^10^ Although a previous meta-analysis by Stubbs *et al* attempted to assess the risk of OSA in BD patients, it had a number of limitations such as a relatively small number of studies including only 12 studies.^11^ This review aimed to sum up the existing body of literature to systematically evaluate the incidence rate and predictors of OSA in BD patients.

## METHODS

Cochrane Handbook (version 5.1.0) guidelines were followed, and the Preferred Reporting Items for Sstematic Reviews and Meta-Analyses (PRISMA) framework was adhered to throughout the review process. Study protocol was registered with PROSPERO (CRD42023484860).^12^,^13^ PubMed, Scopus, CENTRAL (Cochrane Central Register of Controlled Trials), and Google Scholar databases were searched for reports in English published up from 1^st^ January 1960 to 31^st^ October 2023. The search aimed to identify studies reporting the incidence of OSA in individuals with BP and providing sufficient data for quantitative analysis.The key search terms included (“Obstructive Sleep Apnea” OR “OSA”) AND (“Bipolar Disorder” OR “BD”) AND (“Prevalence” OR “Incidence” OR “Epidemiology”) AND (“Predictors” OR “Risk Factors” OR “Associations”) AND (“Anxiety” OR “Depression” OR “Quality of Life”) AND (“Chronic Obstructive Pulmonary Disease” OR “COPD”) AND (“Chronic Bronchitis”) AND (“Sleep Patterns” OR “Sleep Disturbances”). Detailed search strategy have been provided as supplementary Table-SI. Reference lists of selected studies were manually searched for other relevant publications. Duplicate or overlapping studies were cross-checked manually by two authors independently. Any discrepancies were resolved through discussion or consultation with a third author to ensure accurate selection of studies for inclusion in the analysis. Our search was limited to studies involving human subjects.

**Table-I:** Characteristics of included studies investigating for Prevalence and predictors of obstructive sleep apnea in bipolar disorder.

S. No	Author & Year	Country	Study Design	Study Period	Number of BD	OSA Prevalence %	Study Population	M/F	Age (Mean ± SD)	BMI (Mean ± SD)	BD Diagnostic Criteria	OSA Criteria	NOS Quality Score
1	Winkelman et al. 2001^18^	USA	RCS	Sep 1991 - Jun 1996	92	18.5	Referrals for sleep disturbances on psychiatric inpatients	30/62	38.0 ± 15.0	27.9 ± 7.6	DSM-III-R BD-I and BD-II	RDI > 10	4
2	Levine et al. 2001^9^	USA	RCS	1998-1999	66	2.9	Consecutive psychiatric patients at state hospitals	39/27	42 ± 12	NR	DSM-IV bipolar disorder	Overnight PSG and oxyhemoglobin desaturation	5
3	Sharafkhaneh et al. 2005^19^	USA	CS	1992-2001	71362	6.94	Inpatient records of Veteran’s Health Administration from 1992–2001	64621/6741	NR	NR	ICD-9-CM:296.4-296.8	ICD-9-CM: 780.51, 780.53, 780.57	6
4	Hattori et al. 2009^20^	Japan	CS	Sep 1991 - Jun 1996	13	61.5	Mood disorder patients with HAM-D ≥ 10 and clinical sign of OSA	7/6	NR	NR	DSM-IV bipolar affective disorder, HAM-D ≥ 10	AHI ≥ 5	6
5	Soreca et al. 2012^21^	USA	PCS		72	54.1	Patients with bipolar I disorder	30/42	45.30 ± 7.95	36.80 ± 7.69	DSM–IV (SCID–IV)	AHI ≥ 5	5
6	Kelly et al. 2013^10^	USA	RCS	Oct 2005 - Dec 2008	482	21	Consecutive patients at a depression and bipolar clinic	193/289	M: 43.53 ± 15.10; F: 45.37 ± 14.17	NR	BD-I, BD-II, BD-NOS	AHI ≥ 15 or AHI ≥ 5 with EDS	5
7	Bradley et al. 2017^22^	UK	CS		46	29	Outpatients with BD type I or II, in any mood state	15/31	46.8 ± 11.1	30.0 ± 6.7	DSM-IV	AHI ≥ 5	6
8	Fehr et al. 2018^23^	USA	RCS	Aug 2014 - Nov 2014	24	16.6	Inpatient records of VA outpatient with psychiatric disorder	18/6	48.54 ± 13.37	29.63 ± 6.11	DSM-V bipolar disorder	AHI ≥ 5	5
9	Chang et al. 2019^24^	Taiwan	RCS		3650	10.05	Patients with bipolar disorder and comparative controls	1601/2049	39.84 ± 16.55	NR	ICD-9-CM:296.0X-296.8X	ICD-9-CM: 327.23, 780.51, 780.53, 780.57	4
10	Okada et al. 2022^25^	Japan	RCS	Feb 2009 - Mar 2019	43	70	Patients with psychiatric disorders who had undergone attended PSG to examine sleep disorders	25/18	50.6 ± 14.3	26.6 ± 5.4	DSM-IV	AHI ≥ 5	5
11	Patel et al. 2022^26^	USA	CS	2010 - 2014	162	9.3	Patients with major depressive disorder and bipolar disorders	65/97	16.2 ± 1.77	NR	ICD-9	ICD-9	6
12	Merrill et al. 2023^27^ PRINTDATE \* MERGEFORMAT	USA	RCS	2016 - 2020	646	25.23	Mental disorder patient		NR	NR	ICD-10-CM: F31	ICD-10-CM: G47.3	5
13	Drakatos et al. 2023^28^	UK	CS	2015-2019	63	50.8	Patients with psychiatric diagnoses of BD and RDD	31/32	41.8 ± 12.4	32.9 ± 7.98	DSM-V	AHI ≥ 5	7
14	Bonfils et al. 2023^29^	USA	PCS	2011-2019	12486	24	Sleep disorders in veterans with serious mental illnesses	NR	NR	33.1 ± 6.9	ICD-9 & 10	ICD-9 & 10	4

***Abbreviations:*** BD-Bipolar Disorder, OSA-Obstructive Sleep Apnea, PCS-Prospective Cohort Study; RCS-Retrospective Cohort Study; CS- Crsoss Sectional, NR-Not Reported, M-Male, F-Female, RDDRecurrent depressive disorder, ICD- International Classification of Diseases, DSM- Diagnostic and Statistical Manual of Mental Disorders, AHI- Apnea-hypopnea index, NOS-Newcastle Ottawa Scale.

### Inclusion Criteria:

- Population (P).
- Individuals diagnosed with bipolar disorder (BD) of any type.
- Intervention/Exposure (I).
- Studies reporting the prevalence of OSA in individuals with BD.
- Comparator (C).
- Not applicable as this is a prevalence review.
- Outcome (O).
- Primary Outcome: To determine prevalence of OSA in individuals with BD.
- Secondary Outcome: To identify predictors that correlate with the prevalence of OSA.
- Study design (S).
- Observational studies, including cross-sectional, cohort, and case-control designs.

### Exclusion Criteria:

- Studies not reporting relevant data.
- Studies that do not provide relevant information on OSA in BD patients.

### Data Extraction:

Data was independently extracted by the two authors using a standardized form and included: study identifier, sample size for BD groups, participant gender distribution, characteristics of the study population, mean age, mean BMI, risk factors as predictors, BD and OSA diagnostic criteria. Any disagreement between two authors were resolved by discussion with a third author.

### Statistical Analysis:

A meta-analysis was done using the DerSimonian-Laird proportion method (DerSimonian and Laird, 1988), employing STATA version 12.0 (StataCorp LP, College Station, TX), Stats Direct, and Comprehensive Meta-Analysis software (version-3). The pooled prevalence was calculated through inverse-variance-weighted random effects meta-analysis, given the expected heterogeneity in the data. In addition, meta-regression analyses were performed (if N ≥ 4) to explore potential moderators, including age, percentage of males, and body mass index (BMI), using Comprehensive Meta-Analysis software (version-3). This study chose to conduct our analyses only when data from four or more studies were available to ensure robustness and statistical reliability of the results, as smaller datasets may lead to biased or unstable estimates. This approach aligns with recommendations in meta-analytic methodologies to reduce heterogeneity and enhance the credibility of findings.^14^ Sensitivity analysis was done by a single study exclusion method. P<0.05 indicated statistical significance.

### Quality Assessment:

Quality was assessed by the Newcastle–Ottawa Scale (NOS) This scale evaluates studies based on selection, comparability, and exposure factors, with scores from 0 (lowest quality) to 8 (highest quality).^15^ Two authors independently assessed study quality, and a third author was consulted in cases of disagreements. The studies scores 0-3 are assigned to low quality, 4-6 are assigned to moderate quality, and 7-9 are assigned to high quality.^15^

### Publication Bias:

Publication bias was assessed by funnel plot analysis, and Egger’s regression test.^16^,^17^

## RESULTS

**Fig.1:**
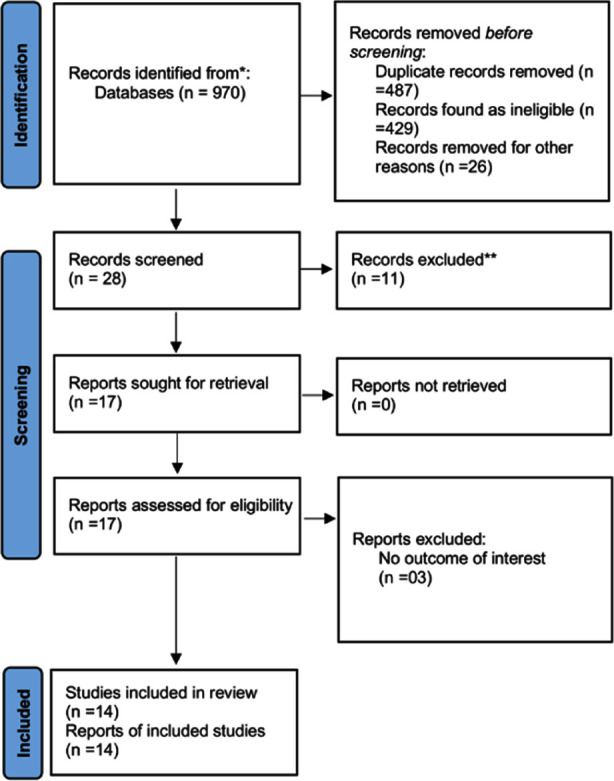
PRISMA 2020 Flow diagram for the selection of studies and specific reasons for exclusion from the present meta-analysis.

Initial search identified 970 references. Of them, 487 were removed as duplicates. After reviewing the titles and abstracts, additional 176 references were excluded as not meeting our inclusion criteria. Full texts of 17 publications were assessed, and 14 studies,^9^,^10^,^18^–^29^ encompassing a total of 89,207 individuals with BD, were used for the final analysis Fig.1.

### Characteristics of the included studies:

All 14 included studies, spanning from 2001 to 2023, focused on Caucasian populations. Two studies had a prospective design, seven were retrospective cohort, and the remaining five were cross-sectional studies.^9^,^10^,^18^–^29^ Average age of study participants was 41.2 ± 9.6 years. Average BMI, reported in seven studies with complete data, was 30.9 ± 3.5 kilograms per square meter (kg/m^2^) indicating an overweight status. Sample sizes varied significantly, ranging from 13 to 71,362 patients diagnosed with BD. Notably, NOS scores were mostly moderate across all studies, ranging from four to six, except one study with high score of seven. This indicates a consistent level of methodological rigor, and suggests that these methodological variations did not significantly impact the observed prevalence of OSA in patients with BD Table-I.

**Table-SI:** Keywords used in the search strategy according to Databases.

***PubMed***:
• ("Obstructive Sleep Apnea"[MeSH] OR “OSA”)
• AND ("Bipolar Disorder"[MeSH] OR “BD”)
• AND ("Prevalence” OR “Incidence” OR “Epidemiology”)
• AND ("Predictors” OR “Risk Factors” OR “Associations”)
• AND ("Anxiety” OR “Depression” OR “Quality of Life”)
• AND ("Chronic Obstructive Pulmonary Disease"[MeSH] OR “COPD”)
• AND ("Chronic Bronchitis"[MeSH])
• AND ("Sleep Patterns” OR “Sleep Disturbances”).
***Scopus***:
• TITLE-ABS-KEY(("Obstructive Sleep Apnea” OR “OSA”)
• AND ("Bipolar Disorder” OR “BD”)
• AND ("Prevalence” OR “Incidence” OR “Epidemiology”)
• AND ("Predictors” OR “Risk Factors” OR “Associations”)
• AND ("Anxiety” OR “Depression” OR “Quality of Life”)
• AND ("COPD” OR “Chronic Obstructive Pulmonary Disease”)
• AND ("Chronic Bronchitis”)
• AND ("Sleep Patterns” OR “Sleep Disturbances”)).
***CENTRAL (Cochrane Central Register of Controlled Trials)***:
• ("Obstructive Sleep Apnea” OR “OSA”)
• AND ("Bipolar Disorder” OR “BD”)
• AND ("Prevalence” OR “Incidence” OR “Epidemiology”)
• AND ("Predictors” OR “Risk Factors” OR “Associations”).
***Google Scholar***:
• “Obstructive Sleep Apnea” OR “OSA”
• “Bipolar Disorder” OR “BD”
• “Prevalence” OR “Incidence” OR “Epidemiology”
• “Predictors” OR “Risk Factors” OR “Associations”
• “Anxiety” OR “Depression” OR “Quality of Life”
• “Chronic Obstructive Pulmonary Disease” OR “COPD”
• “Sleep Patterns” OR “Sleep Disturbances".

Additional Efforts: Reference lists of identified studies were manually reviewed to ensure no relevant publications were overlooked.

### Prevalence of OSA in Bipolar Disorder:

**Fig.2:**
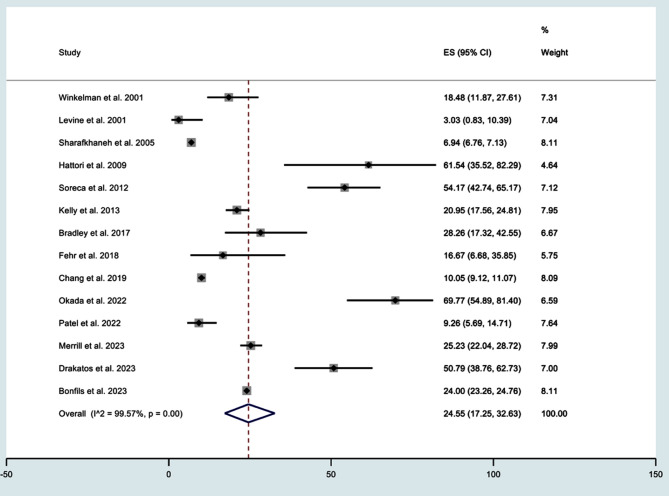
Forest plot for prevalence of obstructive sleep apnea in bipolar disorder.

The analysis of the OSA prevalence encompassed 89,207 individuals diagnosed with BD, and showed that the combined prevalence of OSA was 24.55% (95% confidence interval (CI); 17.25 to 32.63%). This underscores a notable prevalence of OSA within the BD population, as illustrated in Fig.2. However, the findings also revealed a higher level of heterogeneity (I^2^=99.5%), suggesting considerable variability across the included studies.

Subgroup analyses on the rate and predictors of OSA in patients with BD is presented in Table-II. There was a high prevalence of OSA, associated with BD, in North American studies (18.13%, 95% CI: 9.95 to 28.04) with significant heterogeneity (I^2^=99.7%). Retrospective cohort studies demonstrated OSA incidence of 20.70% (95% CI: 12.03 to 30.92) among BD patients. In terms of BD diagnostic criteria, studies using the Diagnostic and Statistical Manual of Mental Disorders (DSM), Fourth Edition (DSM-IV) reported an OSA prevalence of 39.97% (95% CI: 12.89 to 70.68).^30^ In terms of OSA diagnostic criteria, studies using Apnea Hypopnea Index (AHI) demonstrated an OSA prevalence of 41.90% (95% CI: 25.22 to 59.54) associated with AHI) ≥ 5.^31^

**Table-II:** Summary of estimates based on subgroup analysis for Prevalence and predictors of obstructive sleep apnea in bipolar disorder.

Variables	subgroups items	No. of studies	Pooled Prevalence (95% Confidence Interval)	Degree of Heterogeneity	

				I^2^(%)	p* values
Study Location	Asian	3	43.92 (2.97 to 90.88)	-	-
	North American	9	18.13 (9.95 to 28.04)	99.72	<0.001
	European	2	41.04 (31.88 to 50.51)	-	-
Study design	Retrospective cohort	7	20.70 (12.03 to 30.92)	96.91	<0.001
	Cross-sectional	5	26.20 (10.53 to 45.59)	96.35	<0.001
	Prospective cohort	2	23.98 (23.23 to 24.74)	-	-
BD Diagnostic criteria	DSM-III	1	18.48 (11.87 to 27.61)	-	-
	DSM-IV	5	39.97 (12.89 to 70.68)	95.46	<0.001
	ICD-9	3	8.53 (6.01 to 11.44)	-	-
	BD-II	1	20.95 (17.56 to 24.81)	-	-
	DSM-V	2	40.67 (30.39 to 51.36)	-	-
	ICD-10	2	24.05 (23.32 to 24.78)	-	-
OSA Diagnostic criteria	RDI > 10	1	18.48 (11.87 to 27.61)	-	-
	PSG	1	3.03 (0.83 to 10.39)	-	-
	ICD-9	3	8.53 (6.01 to 11.44)	-	-
	AHI ≥ 5	7	41.90 (25.22 to 59.54)	93.00	<0.001

BD-Bipolar Disorder, ICD- International Classification of Diseases, DSM- Diagnostic and Statistical Manual of Mental Disorders, AHI- Apnea-hypopnea index, RDI-Respiratory Disturbance Index, PSG- polysomnography.

### Sensitivity Analysis:

**Fig.3:**
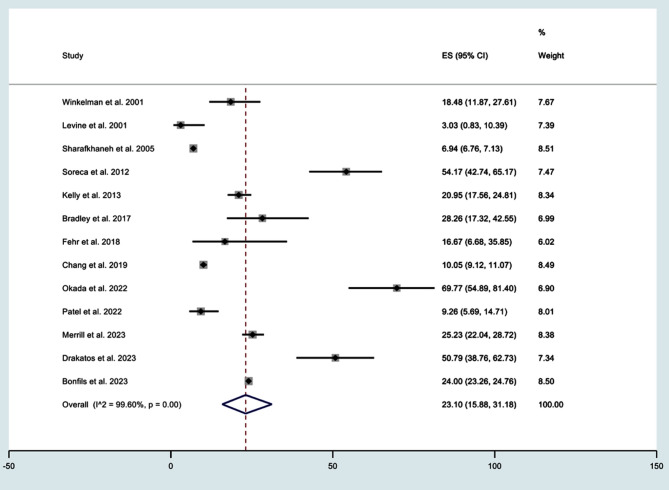
Forest plot after Sensitivity analysis for prevalence of obstructive sleep apnea in bipolar disorder.

As shown in Supplementary Fig.1, sensitivity analyses identified significant impact of one study, conducted by Hattori et al. (2009). After eliminating the study, the recalculated pooled prevalence of OSA was determined based on the remaining studies, encompassing a substantial cohort of 89,194 patients with BD. The revised estimate for the prevalence of OSA was found to be 23.10% (95% CI 15.88 to 31.18). Fig.3 This study also conducted a sensitivity analysis with subgroups based on the NOS score. A study by Hattori et al. was identified as an outlier in this analysis.^20^

### Publication Bias:

Examination of the funnel plot (Supplementary Fig.2) showed that the distribution of included studies around the estimated effect was symmetrical (p=0.09). This symmetry in the pattern implies a well-balanced representation of studies across the entire spectrum of effect sizes. Such equilibrium in distribution serves as an indication that there is no significant presence of publication biases that might distort our results.

### Meta-regression Analyses:

Data from 12 studies were used to explore potential predictors influencing the prevalence of OSA in patients with BD. The analysis indicated that male gender was a significant predictor of a greater incidence of OSA associated with BD (β = 0.30, 95% CI; 0.14 to 0.45, z = 0.07, p = 0.002, R^2^ = 0.57). Meta-regression analysis of seven studies suggested that BMI and mean age had no significance in predicting higher prevalence of OSA (β = 0.15, 95% CI; -1.93 to 2.24, z =0.8, p = 0.85, R^2^ = -20.2, and β = -0.16, 95% CI; -0.85 to 0.51, z =0.29, p = 0.58, R^2^ = 13.48, respectively) Table-III.

**Table-III:** Meta-regression analyses of OSA Prevalence in Patients with BD.

Predictors	No. of Studies	β	95% CI	z	p-value	R^2^
Male Gender	12	0.30	0.14 to 0.45	0.07	0.002	0.57
BMI	7	0.15	-1.93 to 2.24	0.8	0.85	-20.2
Mean Age	7	-0.16	-0.85 to 0.51	0.29	0.58	13.48

## DISCUSSION

Our study indicated a higher prevalence of OSA in patients with BD. This study did not detect evidence of publication biases affecting our results. Meta-regression analyses revealed that male gender was a significant predictor of higher OSA incidence in BD patients, while BMI and mean age showed no significance. Our findings further emphasize the need for additional research and interventions in this population of patients. In 1990, there were 32.7 million cases of bipolar disorder globally, increasing to 48.8 million in 2013-a 49.1% rise attributed to population growth and aging.^32^ In 2013, bipolar disorder accounted for 9.9 million DALYs (0.4% of total disability-adjusted life years (DALYs) and 1.3% of total Years lived with disability (YLDs), with 5.5 million DALYs in females and 4.4 million in males.^33^ DALYs appeared from age 10, peaked in the 20s, and then declined, remaining relatively constant across different regions.^32^ In a prior meta-analysis involving 570,121 patients with severe mental illness (SMI), the reported OSA prevalence was 25.7%, which is similar to our study.^11^

Many patients diagnosed with BD have a convergence of multiple risk factors for OSA,^34^–^36^ such as elevated susceptibility to somatic diseases, particularly cardiovascular diseases.^37^,^38^ Studies consistently report that most patients with sleep apnea have a BMI exceeding 30, underscoring obesity as a recognized risk factor for OSA.^39^ In our analysis, however, only seven studies provided data on BMI, and information on other significant risk factors-such as type II diabetes, essential hypertension, dyslipidaemia, and CVD-was notably absent.^18^,^21^–^23^,^25^,^28^,^29^ BMI’s association with OSA could be diluted due to differences in baseline BMI ranges, non-linear threshold effects, or confounding variables like neck circumference and central obesity. Similarly, age-related OSA risk may be influenced by factors like hormonal changes, comorbidities, and cumulative effects of other predictors, which might obscure its independent contribution. These findings highlight the need for more standardized data collection and subgroup analyses to better elucidate these relationships.

Several studies have highlighted a significant prevalence of OSA among individuals with psychiatric disorders.^40^–^42^ Notably, patients with conditions such as post-traumatic stress disorder (PTSD) and BD show a higher prevalence of OSA compared to the general population.^43^ On the other hand, individuals diagnosed with OSA are also reported to have higher incidences of psychiatric disorders, particularly depression and anxiety.^44^,^45^ These associations suggest a bidirectional relationship, where psychiatric conditions and OSA may influence each other’s onset and progression. Despite increasing prevalence of OSA in the general population and greater awareness among clinicians, no special attention is paid to OSA in association with BD. Medications frequently prescribed for BD management, including benzodiazepines and atypical antipsychotics, carry the potential to exacerbate sleep-related breathing disorders.^46^,^47^

Patients with BD may be at higher risk for OSA due to the side effects of common treatments. Medications like atypical antipsychotics (e.g., olanzapine, quetiapine, risperidone) and mood stabilizers (e.g., valproic acid, lithium) often cause significant weight gain, a key risk factor for OSA. These drugs can also exacerbate OSA by inducing muscle relaxation or impairing arousal responses to airway obstruction during sleep. The bidirectional relationship between OSA and BD, wherein sleep apnea worsens mood instability and vice versa, underscores the need for routine OSA screening and integrated management in BD patients to improve both sleep and psychiatric outcomes. Prolonged use of mood stabilizers and other medications associated with BD treatment may elevate the risk of apnea through diverse mechanisms, including but not limited to weight gain, diminished arousability during sleep, and dysfunction of upper airway muscles.^18^ Consequently, it becomes imperative for clinicians to perform a thorough evaluation for OSA before contemplating the initiation of such pharmacological treatments.

The variation in reported prevalence rates of OSA in BD patients in our study may be attributed to differences in the definitions of clinical samples among the included reports. For instance, Hattori et al. noted a high prevalence of 69%, attributed to the inclusion of patients with active depressive symptoms and clinical signs of OSA.^20^ In contrast, Levine et al.^9^ reported a lower rate of 2.9%, as their study exclusively recruited patients without pre-existing sleep conditions. A sole population cohort study by Sharafkhaneh et al. demonstrated that the rate of OSA in patients with BD was 6.94%.^19^ Previous meta-analysis concluded that there was insufficient evidence of increased occurrence of OSA in patients with BD.^11^

The observed link between BD and the risk of OSA may be explained by several mechanisms. Firstly, various types of sleep disturbances are described in all phases of BD, including remission.^22^ Sympathetic hyperactivity and hyperarousal states associated with sleep disturbances may destabilize the upper respiratory tract, leading to OSA.^48^ Secondly, benzodiazepines, commonly prescribed for sleep problems in BD, induce muscle relaxation, potentially leading to airway collapse, a defining feature of OSA.^40^ Lastly, the hypothesized link between BD and OSA may be related to the metabolic syndrome.^49^ OSA is often associated with metabolic syndrome symptoms, that, together with often limited access to quality physical health care, may negatively impact patients outcomes.^50^ Medications, including lithium carbonate or antiepileptic drugs often combined with atypical antipsychotic drugs, may further contribute to this predisposition.^49^

The strength of our study lies in its robust methodology, including a comprehensive meta-analysis that incorporated a large sample size and rigorous statistical techniques, such as meta-regression. By identifying male gender as a significant predictor of OSA in patients with BD, our findings provide reliable and clinically relevant insights. Our study revealed a higher incidence of OSA in males compared to females, with the risk trend primarily attributed to male BD patients. This study may speculate that women may experience heightened activity in the dilator muscles of the upper respiratory tract, reducing the likelihood of upper airway closure during sleep.^51^ Moreover, men tend to have greater fat deposition in the upper respiratory tract, compared to women. Additionally, variations in respiratory control between genders, potentially influenced by hormones, may also contribute to these observed differences. Further research is needed to explore the underlying mechanisms, offering insights for targeted interventions in this intricate intersection of psychiatric and sleep disorders.

### Limitations:

First, the limited number of available studies investigating the coexistence of OSA and BD impedes the depth of analysis and generalizability of our results. The small pool of studies investigating the coexistence of OSA and BD limits the depth of analysis and generalizability of our results. The included studies exhibit methodological diversity, encompassing prospective, retrospective, and cross-sectional designs. This heterogeneity in data collection and reporting introduces significant variability, impacting the consistency of our findings. Additionally, substantial variability in sample sizes across selected studies may impact the statistical power and reliability of the meta-analysis.

The temporal span of publication years (2001 to 2023) introduces potential variations in diagnostic criteria, treatment approaches, and prevalence rates. Geographic differences also pose a factor that could influence the applicability of findings to diverse populations. None of included studies provided data on risk factors or comorbidities for OSA in BD beyond gender, BMI and age as a predictive factor. As such, it was not possible to include risk factor information in our analysis.

## CONCLUSION

The study found a higher incidence of OSA among patients with BD. Male gender emerged as a significant predictor of OSA, while BMI and mean age did not show a significant association in this population. These findings highlight the importance of recognizing the comorbidity of BD and OSA in clinical practice. Tailoring interventions based on key predictors may enhance the management and care of individuals affected by both conditions.

### Authors’ contributions:

**FZ:** Conceived and designed the study.

**CC** and **KG:** Collected the data and performed the analysis.

**FZ:** Was involved in the writing of the manuscript and is responsible for the integrity of the study.

All authors have read and approved the final manuscript.

***Note:*** Information Regarding Supplementary Figures can be Obtained from the correspondence author.
